# RP11-295G20.2 facilitates hepatocellular carcinoma progression via the miR-6884-3p/CCNB1 pathway

**DOI:** 10.18632/aging.103552

**Published:** 2020-07-20

**Authors:** Jing Li, Tingting Xia, Junyan Cao, Donghong He, Zhaocong Chen, Biao Liang, Jie Song

**Affiliations:** 1 Center of Digestive Endoscopy, Guangdong Second Provincial General Hospital, Guangzhou 510317, China; 2 Integrated Chinese and Western Medicine Postdoctoral Research Station, Jinan University, Guangzhou 510632, China; 3 Center for Reproductive Medicine, The Third Affiliated Hospital of Sun Yat-Sen University, Guangzhou 510630, Guangdong, China; 4 Department of Medical Ultrasonic, The Third Affiliated Hospital of Sun Yat-Sen University, Guangzhou 510630, Guangdong, China; 5 Department of Rehabilitation Medicine, The Third Affiliated Hospital of Sun Yat-Sen University, Guangzhou 510630, Guangdong, China

**Keywords:** hepatocellular carcinoma, RP11-295G20.2, miR-6884-3p, CCNB1

## Abstract

Objective: An increasing number of studies have indicated that long noncoding RNAs (lncRNAs) play an important role in the pathogenesis of hepatocellular carcinoma (HCC). In this study, we aimed to clarify the roles of RP11-295G20.2 in HCC progression and the underlying molecular mechanisms.

Results: Bioinformatics analyses based TCGA data suggested that RP11-295G20.2 was significantly upregulated in HCC tissues and increased RP11-295G20.2 expression level correlated with poor overall survival of patients with HCC. The results of RT-PCR further showed that RP11-295G20.2 was upregulated in HCC tissues and cell lines. Functionally, RP11-295G20.2 knockdown significantly inhibited the proliferation, colony formation, invasion and migration, but induced the apoptosis of HCC cells. In line with this, downregulation of RP11-295G20.2 in HCC lines markedly suppressed the tumor growth *in vivo*. Mechanistically, RP11-295G20.2 could upregulate CCNB1 through targeting miR-6884-3p. More importantly, our rescue experiments revealed that miR-6884-3p/CCNB1 axis was involved in RP11-295G20.2-meditated tumorigenic behaviors of HCC cells.

Conclusions: RP11-295G20.2 can contribute to HCC progression at least partly via the miR-6884-3p/CCNB1 axis, suggesting that RP11-295G20.2 may be a potential target for HCC therapy.

Methods: RT-qPCR was employed to examine the expression levels of RP11-295G20.2, miR-6884-3p, and CCNB1 in HCC tissues and cell lines. CCK8 assay, transwell assay, colony formation assay and flow cytometry analysis were performed to evaluate the biological function of RP11-295G20.2 in HCC cells. The xenograft tumor assay was used to assess the effect of RP11-295G20.2 on the in vivo growth of HCC cells. The luciferase reporter assay, RIP assay and Spearman's correlation analysis were performed to explore the potential mechanisms underlying the roles of RP11-295G20.2 in HCC progression.

## INTRODUCTION

Hepatocellular carcinoma (HCC) is one of the major causes of cancer-associated death worldwide [1, 2]. HCC is characterized by the high susceptibility to recurrence and metastasis. Up to date, the long-term survival of patients with HCC remains discouraging, though substantial progression in surgical therapy, interventional therapy, radiation and chemotherapy have been made during the past decades [3–5]. Therefore, it is still imperative to further elucidate the mechanisms underlying HCC progression to identify the therapeutic targets for HCC.

Long noncoding RNAs (lncRNAs) are a class of endogenous RNAs in length with 200 or more nucleotides, which are featured with no or limited protein-coding capacities [6, 7]. LncRNAs are implicated in many cellular biological processes, such as cell differentiation, epigenetic regulation, and genomic imprinting [8, 9]. Mounting evidence shows that expression profiles of lncRNAs are dysregulated in a variety of cancers and play an essential role in accelerating cancer progression via multiple molecular mechanisms [9, 10]. Zhang et al. demonstrated that overexpression of lncRNA HULC could promote the proliferation, migration, and invasion of HCC cells *in vitro*, as well as xenograft tumor growth *in vivo* through targeting miR-2052 as a competing endogenous RNA (ceRNA) [11] Chen et al. suggested that lncRNA CDKN2B-AS expression was able to facilitate HCC metastasis via the miR-153-5p/ARHGAP18 axis [12]. Cheng et al. revealed that lncRNA HOTAIR could epigenetically suppress the miR-122 expression by inducing its DNA methylation, which led to aberrant upregulation of cyclin G1 expression, ultimately promoting HCC progression [13]. Qin et al. found that lncRNA PSTAR could enhance the interaction between hnRNP K and p53 via the SUMOylation of heterogeneous nuclear ribonucleoprotein K (hnRNP K) to inactivate p53, which largely accelerated HCC progression [14]. Collectively, lncRNAs may be exploited as therapeutic targets for HCC due to their involvement in tumor progression. RP11-295G20.2 is a newly identified lncRNA and its expression pattern and biological functions in HCC have not been explored yet. Therefore, in this study we attempted to elucidate the roles of RP11-295G20.2 in HCC progression and the relevant molecular mechanisms by the *in vitro* and *in vivo* experiments, so as to provide the theoretical basis for developing the potential targets for HCC treatment.

## RESULTS

### RP11-295G20.2 is overexpressed in HCC and correlates with poor prognosis

**Figure 1:**
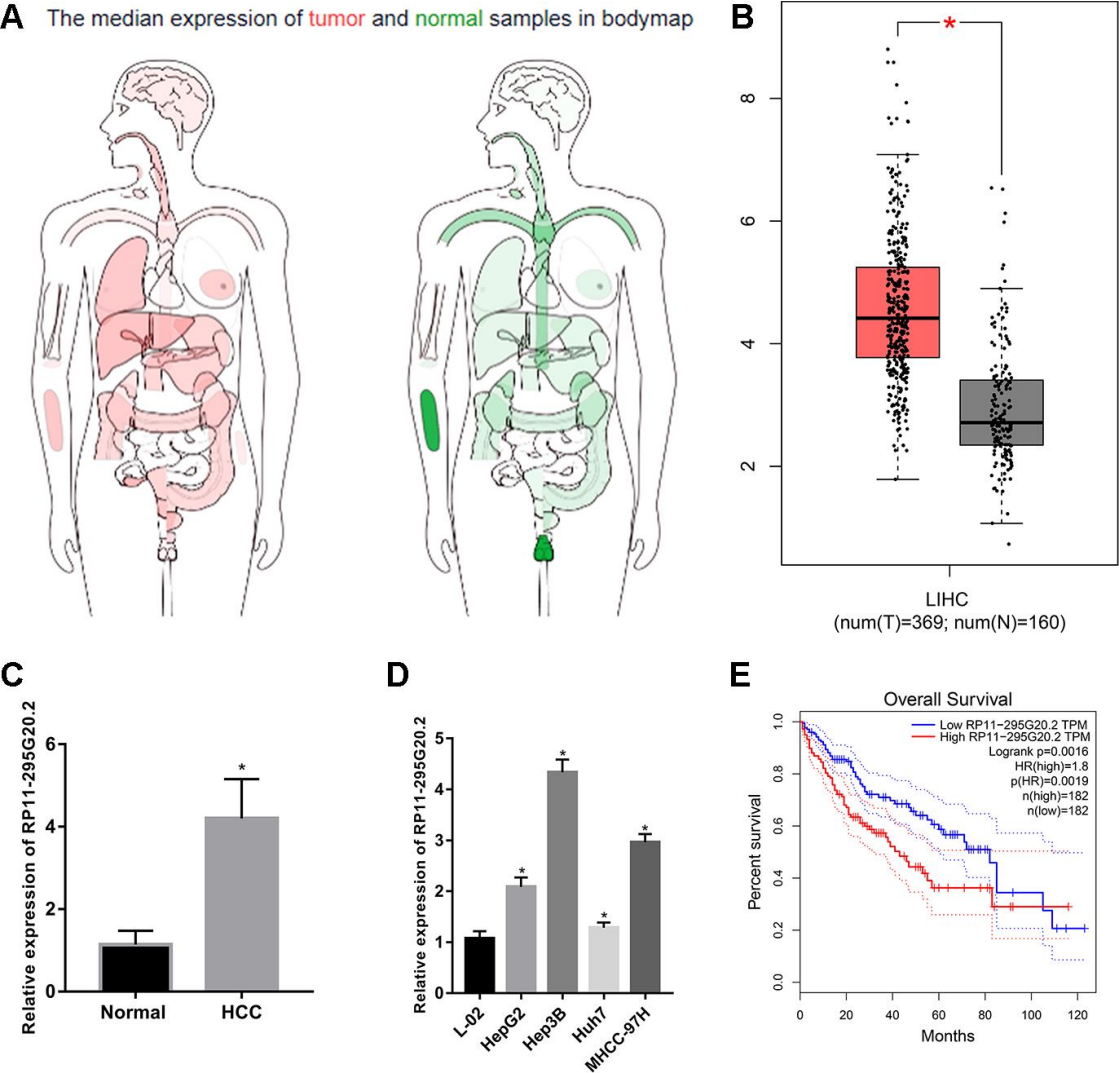
**RP11-295G20.2 is highly expressed in HCC tissues and cell lines.** (**A**) The expression profile of RP11-295G20.2 in various cancers by analysing TCGA database using GEPIA. (**B**) The expression level of RP11-295G20.2 in HCC by analysing GEPIA, *P < 0.05. (**C**) RT-qPCR results of RP11-295G20.2 expression in HCC tissues (n=30) and paracancerous tissues(n=30), *P < 0.05. (**D**) RP11-295G20.2 expression in HCC cell lines was detected using RT-qPCR, *P < 0.05. (**E**) Kaplan-Meier analysis of the correlation between RP11-295G20.2 expression and overall survival (OS) in patients with HCC using GEPIA.

We evaluated the RP11-295G20.2 expression patterns in HCC and normal adjacent liver tissues using The Cancer Genome Atlas (TCGA) data through GEPIA [15]. The result showed that RP11-295G20.2 was upregulated in HCC tissues versus adjacent normal tissues (Figure 1A, 1B). Then, we performed RT-qPCR assay to further analyze the RP11-295G20.2 expressions in 30 pairs of HCC samples and adjacent normal liver tissue samples from our center, as well as in HCC cell lines (HepG2, Hep3B, Huh7 and MHCC-97H) and normal liver cell line (L0-2). Consistently, we found that RP11-295G20.2 expression level is significantly increased in HCC tissues (Figure 1C) and cell lines (Figure 1D) compared with that in normal liver tissues and cell line. Furthermore, bioinformatics analysis based on TCGA data showed that there was a positive correlation between higher RP11-295G20.2 expression and shorter overall survival (OS) (Figure 1E).

### RP11-295G20.2 knockdown inhibits the growth, migration and invasion of HCC cells *in vitro*

**Figure 2:**
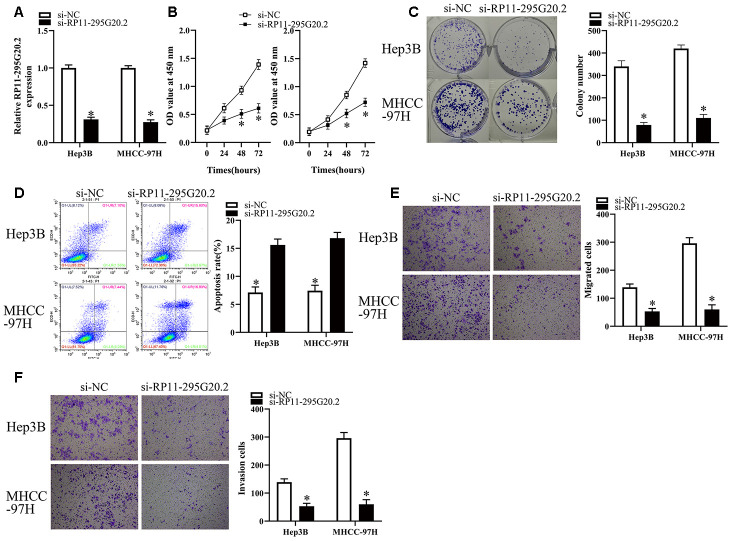
**RP11-295G20.2 knockdown inhibits the growth, migration and invasion of HCC cells.** (**A**) RT-qPCR results indicated RP11-295G20.2 was successfully silenced in Hep3B and MHCC-97H cells, *P < 0.05. The effect of the RP11-295G20.2 knockdown on cell proliferative capacity was examined using CCK-8 (**B**) and colony formation assays (**C**) in Hep3B and MHCC-97H cells, *P < 0.05. (**D**) The cell apoptosis assay showed that the RP11-295G20.2 knockdown markedly increase apoptosis in Hep3B and MHCC-97H cells, *P < 0.05. Transwell migration (**E**) and invasion assays (**F**) were performed to determine the effect of RP11-295G20.2 knockdown on the migration and invasion of Hep3B and MHCC-97H cells, *P < 0.05.

Considering the higher RP11-295G20.2 expression levels in Hep3B and MHCC-97H cells than those in HepG2 and Huh7 cells, we transfected Hep3B and MHCC-97H cells with si-RP11-295G20.2 or si-NC to explore the biological functions of RP11-295G20.2 in HCC. As Figure 2A showed, RP11-295G20.2 was successfully silenced in HCC cells. The impact of RP11-295G20.2 knockdown on cell proliferative capacity was examined using CCK-8 and colony formation assays. The data suggested that RP11-295G20.2 knockdown significantly inhibited the Hep3B and MHCC-97H cell proliferation (Figure 2B, 2C). Then, we explored whether RP11-295G20.2 can regulate HCC cell apoptosis. The apoptosis assay showed that RP11-295G20.2 knockdown markedly increased the apoptosis of Hep3B and MHCC-97H cells (Figure 2D). Additionally, we conducted transwell migration and invasion assays to determine the impact of RP11-295G20.2 knockdown on the migration and invasion of Hep3B and MHCC-97H cells. As illustrated in Figure 2E, 2F, RP11-295G20.2 knockdown significantly impeded the migration and invasion of Hep3B and MHCC-97H cells. Collectively, these results suggested that RP11-295G20.2 knockdown could inhibit the growth, migration and invasion of HCC cells *in vitro*.

### RP11-295G20.2 serves as a competitive endogenous RNA (ceRNA) for miR-6884-3p in HCC cells

**Figure 3:**
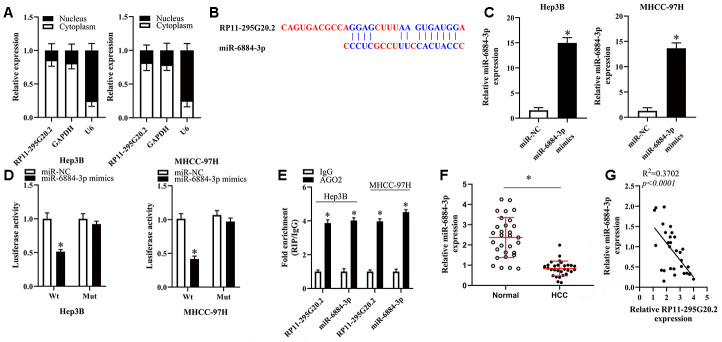
**RP11-295G20.2 sponged miR-6884-3p in HCC cells.** (**A**). RT-qPCR assays in nuclear and cytoplasmic RNA fractions detected the RP11-295G20.2 level in cytoplasm and nuclear. (**B**) The binding sites of miR-6884-3p on the RP11-295G20.2 using Starbase. (**C**) The expression level of miR-6884-3p was detected using RT-qPCR after transfecting miR-6884-3p mimics, *P < 0.05. (**D**) The luciferase reporter assays were performed to measure the luciferase activities of RP11-295G20.2 in response to miR-6884-3p mimics, *P < 0.05. (**E**) RIP followed by RT-qPCR to detect miR-455-3p and RP11-295G20.2 in HCC cells. (**F**) The expression of miR-6884-3p was measured in HCC tissues and normal tissues,*P < 0.05. (**G**) The correlation of miR-6884-3p and RP11-295G20.2 expression in HCC tissues was negative.

To elucidate the potential mechanisms underlying the roles of RP11-295G20.2 in HCC cells, we firstly conducted the nuclear/cytoplasmic fractionation assay to determine the subcellular location of RP11-295G20.2 and found that RP11-295G20.2 was primarily expressed in the cytoplasm of HCC cells (Figure 3A). Solid evidence demonstrates that lncRNAs located in cytoplasm can function as a ceRNA for microRNAs (miRNAs) [16, 17]. Therefore, we hypothesized that RP11-295G20.2 may promote the malignant phenotypes of HCC cells by targeting specific miRNAs. To test this hypothesis, we performed bioinformatics analysis using LncBase v.2 and RNAhybrid to identify the potential miRNAs targeted by RP11-295G20.2 [18, 19]. Using the bioinformatics tool database, we predicted that there are several miRNAs that potentially bind to RP11-295G20.2. Of these, we identified that miR-6817-5p, miR-432-3p, miR-6740-5p, miR-510-5p, miR-6884-3p, miR-6731-5p, miR-625-5p, and miR-876-3p may be the most potential binding targets for RP11-295G20.2. Accordingly, RT-qPCR revealed that miR-6884-3p exhibited the greatest change, so we selected miR-6884-3p for further analysis (Supplementary Figure 1). The bioinformatics prediction suggested that RP11-295G20.2 might exist a binding site for miR-6884-3p (Figure 3B). To validate this putative result, we transfected Hep3B and MHCC-97H cells with RP11-295G20.2-Wt or RP11-295G20.2-Mut coupled with miR-6884-3p mimics or miR-NC for luciferase reporter assay. As shown in Figure 3C, miR-6884-3p mimics were successfully transfected into Hep3B and MHCC-97H cells. Meanwhile, we observed that transfection of miR-6884-3p mimics caused a shark decrease in the luciferase activity of RP11-295G20.2-Wt compared with miR-NC, whereas the luciferase activity of RP11-295G20.2-Mut was not affected by the cotransfection of miR-6884-3p mimics or miR-NC (Figure 3D). Then, we conducted RIP assay to determine whether RP11-295G20.2 really interacts with miR-6884-3p in HCC cells. The data presented in Figure 3E showed that both RP11-295G20.2 and miR-6884-3p were abundantly immunoprecipitated from the lysates of Hep3B and MHCC-97H cells by anti-AGO2 antibody. In accordance with results in HCC cell lines, the miR-6884-3p expression was downregulated in HCC tissues versus in adjacent normal liver tissues (Figure 3F). Moreover, there was an inverse correlation between the miR-6884-3p expression and the RP11-295G20.2 expression (Figure 3G). Taken together, these findings demonstrated that RP11-295G20.2 might function as ceRNA for miR-6884-3p in HCC cells.

### miR-6884-3p inhibits the growth, migration and invasion of HCC cells *in vitro*

**Figure 4:**
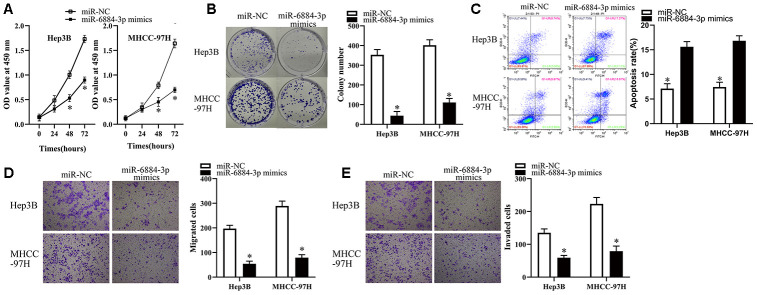
**miR-6884-3p overexpression inhibits the growth, migration and invasion of HCC cells.** (**A**, **B**) Hep3B and MHCC-97H cells transfected with the miR-6884-3p mimics or miR-NC were collected. These cells were subjected to CCK-8 and colony formation assays to determine cell proliferation and colony formation capacity, respectively. *P < 0.05 vs. the miR-NC group. (**C**) Flow-cytometric determination of apoptotic rate of Hep3B and MHCC-97H cells after miR-6884-3p overexpression. *P < 0.05 vs. group miR-NC. (**D**, **E**) Transwell migration and invasion assays were used to the effect of miR-625 overexpression on the migration and invasion of Hep3B and MHCC-97H cells, respectively. Representative images and quantification are presented. *P < 0.05 vs. group miR-NC.

To explore the functions of miR-6884-3p in HCC cells, we first transfected Hep3B and MHCC-97H cells with miR-6884-3p mimics or miR-NC, and then subjected these cells for functional experiments. As illustrated in Figure 4A–4C, miR-6884-3p mimics could significantly inhibit the proliferation and colony formation, but promote the apoptosis of HCC cells. Additionally, transwell migration and invasion assays showed that miR-6884-3p mimics markedly suppressed the migration and invasion of HCC cells *in vitro* versus miR-NC (Figure 4D, 4E). Overall, these results suggested that miR-6884-3p may function as a tumor suppressor in HCC.

### CCNB1 is a direct target gene of miR-6884-3p in HCC cells

**Figure 5:**
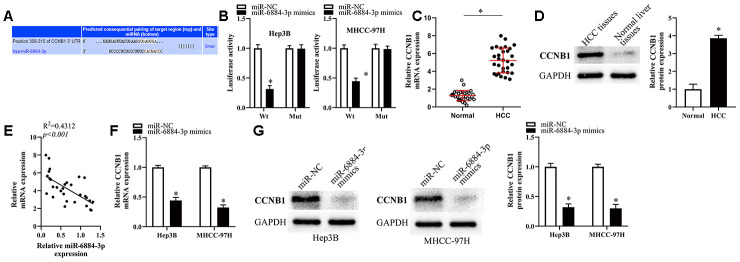
**CCNB1 was the target of miR-6884-3p.** (**A**) Targetscan analysis result showed that miR-6884-3p had a binding site with CCNB1. (**B**) Dual-luciferase reporter gene assay was used to confirm the target relationship between miR-6884-3p and CCNB1 in Hep3B and MHCC-97H cells. RT-qPCR (**C**) and western blotting (**D**) were used to determine the expression of CCNB1 in HCC tissues and adjacent tissues; (**E**) The expression relationship between miR-6884-3p and CCNB1 was evaluated by Spearman’s correlation analysis. (**F**, **G**) The expression of CCNB1 was detected by RT-qPCR and western blotting in Hep3B and MHCC-97H cells,*p<0.05.

To reveal the potential mechanism underlying the effect of miR-6884-3p on HCC cells, we first conducted bioinformatics analysis to screen its putative targets using Targetscan [20] and found that CCNB1 might be a target of miR-6884-3p (Figure 5A). Therefore, we utilized luciferase reporter assays to determine whether CCNB1 is a target of miR-6884-3p in HCC cells. As Figure 5B showed, miR-6884-3p mimics could significantly reduce the luciferase activity of CCNB1 wild type reporter in Hep3B and MHCC-97H cells, whereas the luciferase activity of CCNB1 mutant reporter was not affected by miR-6884-3p mimics. The result indicated that CCNB1 might be a target of miR-6884-3p. The previous studies suggested that CCNB1 was aberrantly upregulated in HCC tissues and played a promotive role in HCC progression [21, 22]. Consistently, we also found that CCNB1 was significantly upregulated in HCC tissues versus in adjacent normal liver tissues (Figure 5C, 5D). In addition, our data showed that miR-6884-3p expression level inversely correlated with CCNB1 mRNA expression level in HCC tissue (Figure 5E). Furthermore, the results of RT-qPCR and western blot showed that the ectopic overexpression of miR-6884-3p dramatically downregulated CCNB1 in HCC cells (Figure 5F, 5G). Taken together, these results demonstrated that CCNB1 may be a direct target of miR-6884-3p in HCC cells.

### Restoration of CCNB1 expression attenuates the tumor-suppressive effects of miR-6884-3p overexpression in HCC cells

**Figure 6:**
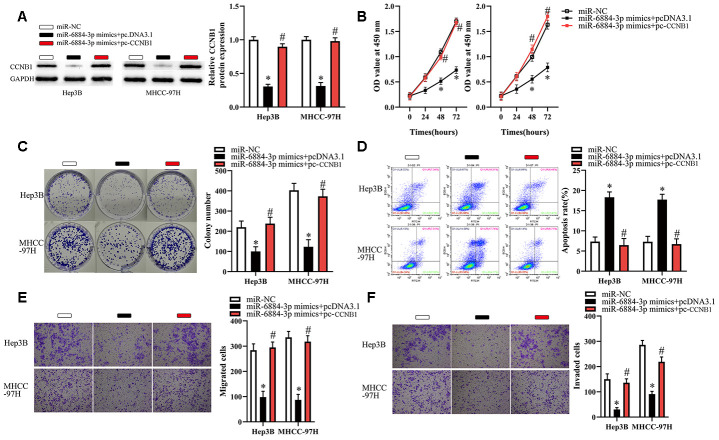
**CCNB1 overexpression partially reverses the effects of miR-6884-3p on Hep3B and MHCC-97H cells.** Hep3B and MHCC-97H cells transfected with the miR-6884-3p mimics with pcDNA3.1 or pc-CCNB1 were subjected to the following assays. (**A**) Western blotting was used to detect the protein level of CCNB1,*P < 0.05. (**B**–**F**) The CCK-8 assay, colony formation assay, cell apoptosis assay, and Transwell migration and invasion assays were performed to test the proliferation, colony formation, apoptosis, migration, and invasion of Hep3B and MHCC-97H cells, respectively,*P < 0.05.

To restore CCNB1 expression, we transfected Hep3B and MHCC-97H cells with miR-6884-3p mimics combined with pcDNA3.1 or pc-CCNB1 without the 3′-UTR. As illustrated in Figure 6A, the miR-6884-3p-meditated downregulation of CCNB1 was successfully attenuated by transfection of pc-CCNB1. Then, we performed a series of functional experiments to explore whether restoration of CCNB1 expression affected the miR-6884-3p-meditated tumor-suppressive effects on HCC cells. As a result, we observed that restoration of CCNB1 expression significantly abrogated the miR-6884-3p-meditated effect on the proliferation, colony formation and apoptosis of HCC cells (Figure 6B–6D). Similarly, our data showed that restoration of CCNB1 expression dramatically reversed the miR-6884-3p-meditated inhibitory effect on the migration and invasion of HCC cells *in vitro* (Figure 6E, 6F). Taken together, these results suggested that miR-6884-3p could inhibit HCC progression at least partly by targeting CCNB1 expression.

### RP11-295G20.2 promotes the growth, migration and invasion of HCC cells via the miR-6884-3p/CCNB1 pathway

**Figure 7:**
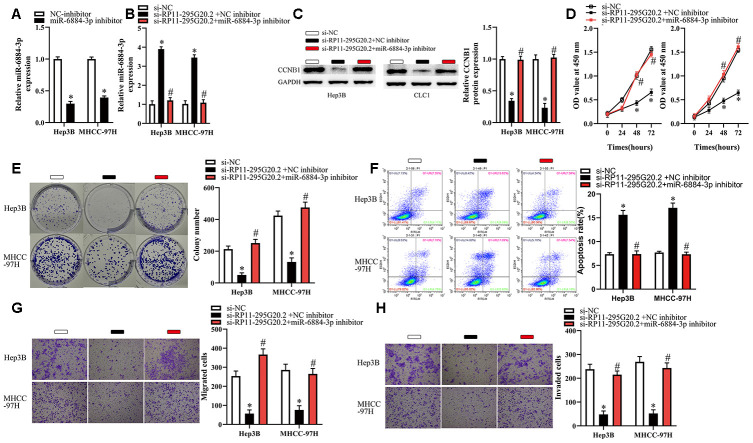
**Inhibition of miR-6884-3p partially reverses the effects of RP11-295G20.2 silence on Hep3B and MHCC-97H cells.** Hep3B and MHCC-97H cells transfected with the si-RP11-295G20.2 with NC inhibitor or miR-6884-3p inhibitor were subjected to the following assays. (**A**, **B**) RT-qPCR was used to detect the protein level of miR-6884-3p,*P < 0.05. (**C**) Western blotting was used to detect the protein level of CCNB1,*P < 0.05. (**D**–**H**) The CCK-8 assay, colony formation assay, cell apoptosis assay, and transwell migration and invasion assays were performed to test the proliferation, colony formation, apoptosis, migration, and invasion of Hep3B and MHCC-97H cells, respectively,*P < 0.05.

To determine whether the miR-6884-3p/CCNB1 pathway meditated the biological functions of RP11-295G20.2 in Hep3B and MHCC-97H cells, we treated HCC cells with si-RP11-295G20.2 and (or) miR-6884-3p inhibitor. As shown in Figure 7A–7C, miR-6884-3p inhibitor was effectively transfected into HCC cells, and si-RP11-295G20.2-induced upregulation of miR-6884-3p and downregulation of CCNB1 were significantly reversed by miR-6884-3p inhibitor. Additionally, miR-6884-3p inhibitor markedly abrogated the effects of si-RP11-295G20.2 on the proliferation, colony formation and apoptosis of HCC cells (Figure 7D–7F). Similarly, transwell migration and invasion assays showed that the miR-6884-3p inhibitor could dramatically mitigated the si-RP11-295G20.2-meditated inhibitory effect on the migration and invasion *in vitro* of HCC cells (Figure 7G, 7H). Taken together, these results suggested that the miR-6884-3p/CCNB1 pathway is involved in RP11-295G20.2-meditated HCC progression.

### RP11-295G20.2 knockdown suppresses HCC growth in vivo

**Figure 8:**
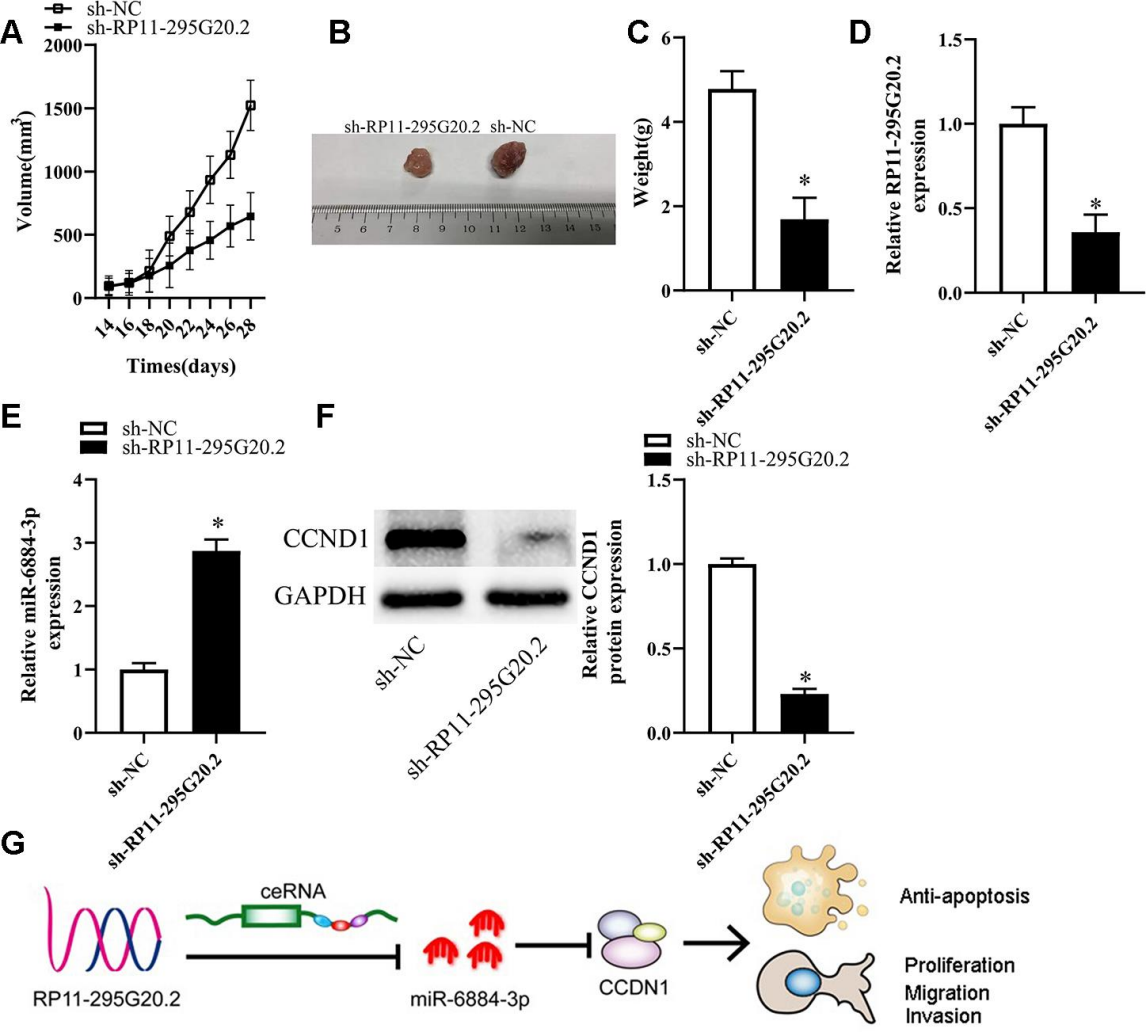
**Knockdown of RP11-295G20.2 suppresses tumor growth in vivo.** (**A**) Quantitative analysis of the average tumour volume derived from si-RP11-295G20.2–transfected or si-NC–transfected HCC cells,*P < 0.05. (**B**) Representative images of tumours derived from the nude mice injected with si-RP11-295G20.2–transfected or si-NC–transfected HCC cells,*P < 0.05. (**C**) Quantitative analysis of the average tumour weights derived from si-RP11-295G20.2–transfected or si-NC–transfected HCC cells,*P < 0.05. (**D**, **E**) The relative expression of RP11-295G20.2, miR-6884-3p, and CCNB1 were detected using RT-qPCR (**D**, **E**) and western blot (**F**), *P < 0.05. (**G**) The mechanism hypothesis of RP11-295G20.2/miR-6884-3p/CCNB1 pathway in HCC.

To clarify the functions of RP11-295G20.2 in vivo, we injected HCC cells transfected with either sh-RP11-295G20.2 or sh-NC into nude mice subcutaneously. As a result, we observed that the tumor volume increased much slower in sh-RP11-295G20.2 group than that in sh-NC group (Figure 8A, 8B). Xenografts were excised from BALB/c nude mice 4 weeks after injection and weighed. As illustrated in Figure 8C, the weight of xenografts was significantly slighter in sh-RP11-295G20.2 group than that in sh-NC group. Meanwhile, we found that both RP11-295G20.2 and CCNB1 were downregulated, whereas miR-6884-3p expression was significantly increased in the tumor xenografts derived from HCC cells transfected with sh-RP11-295G20.2 compared with that in those transfected with sh-NC (Figure 8D–8F).

## DISCUSSION

Although a variety of strategies such as surgical therapy, interventional therapy, radiation and chemotherapy are available for HCC treatment, the long-term survival of HCC patients remains unsatisfying. Therefore, further research is needed to develop the promising targets for HCC therapy to improve the prognosis of HCC patients. In this study, we attempted to clarify the expression profile and biological functions of RP11-295G20.2 in HCC and the underlying mechanisms. Overall, our findings revealed an essential involvement of the RP11-295G20.2/miR-6884-3p/CCNB1 pathway in HCC progression (Figure 8G).

Abnormally expressed lncRNAs can promote or inhibit tumor progression through targeting the tumor-associated miRNAs, indicating that they may be developed as targets for anti-tumor therapy [23, 24]. In this study, for the first time we found that RP11-295G20.2 was significantly upregulated in HCC tissue and cell lines and increased RP11-295G20.2 expression correlated with shorter overall survival of patients with HCC. Meanwhile, we found that RP11-295G20.2 knockdown could significantly inhibit the proliferation, colony formation, migration and invasion, but induce the apoptosis of HCC cells, indicating an oncogenic role of RP11-295G20.2 in HCC progression. Evidence shows that the functions of lncRNAs closely correlate with their subcellular locations [25–27]. To clarify the potential mechanisms for the oncogenic role of RP11-295G20.2 in HCC, we conducted the nuclear/cytoplasmic fractionation assay to determine the subcellular location of RP11-295G20.2 in HCC cells. Our data showed that RP11-295G20.2 was primarily expressed in the cytoplasm of HCC cells, which suggested that RP11-295G20.2 might serve as a ceRNA for miRNAs. Strikingly, we found that RP11-295G20.2 could bind with and inhibited miR-6884-3p in HCC cells. Previous studies indicated that miRNAs play crucial parts in HCC progression [28–30]. However, the roles of miR-6884-3p in tumor progression have never been explored yet. Remarkably, in this study we demonstrated that miR-6884-3p was downregulated in HCC tissues compared with that in adjacent normal liver tissues. Meanwhile, miR-6884-3p mimics could significantly retard the proliferation and colony formation, migration, invasion, but promote the apoptosis of HCC cells. More importantly, miR-6884-3p inhibitor markedly abrogated the effects of si-RP11-295G20.2 on the proliferation, colony formation and apoptosis of HCC cells. Taken together, these results suggested that RP11-295G20.2 might facilitate HCC progression at least partly by targeting miR-6884-3p.

A large amount of evidence confirms that miRNAs can suppress gene express via the direct interaction with the 3′-UTRs of their target mRNAs [29–32]. To elucidate the mechanisms underlying the suppressive effect of miR-6884-3p on HCC progression, we first performed bioinformatics analysis to screen the mRNAs of which the 3′-UTR matches the seed sequence of miR-6884-3p, and then conducted luciferase reporter assays to validate it. Our data showed that there was a pairing region between the 3′-UTR of CCNB1 mRNA and the seed sequence of miR-6884-3p, suggesting that CCNB1 might be a target of miR-6884-3p. The previous studies demonstrated that CCNB1 was aberrantly upregulated and functioned as an oncogene in HCC [33–35]. Therefore, we asked whether miR-6884-3p inhibited HCC progression by targeting CCNB1. To address this question, we first explored the correlation between miR-6884-3p expression level and CCNB1 expression level. As a result, we found that CCNB1 expression level was significantly elevated and inversely correlated with miR-6884-3p expression in HCC tissues. Additionally, our data showed that miR-625 mimics dramatically downregulated CCNB1 expression in HCC cell lines. Furthermore, we demonstrated that restoration of CCNB1 expression markedly abrogated the tumor-suppressive effects of miR-6884-3p in HCC cells. Overall, our data strongly supported the notion that miR-6884-3p may inhibit HCC progression by targeting CCNB1.

As we presented above, RP11-295G20.2 may serve as ceRNA for miR-6884-3p in HCC cells. Thus, we further asked whether RP11-295G20.2 promoted the malignant phenotypes of HCC cells by targeting miR-6884-3p and upregulating CCNB1. Our data showed that RP11-295G20.2 knockdown led to a shark decrease in CCNB1 protein, but miR-6884-3p inhibitor could effectively reversed this effect. On contrast, miR-6884-3p mimics could dramatically attenuate the inhibitory effect of RP11-295G20.2 knockdown on the proliferation, colony formation, migration, and invasion of HCC cells. Similarly, increased apoptosis mediated by RP11-295G20.2 knockdown was also significantly reversed by miR-6884-3p mimics. Taken together, these findings suggested that the miR-6884-3p/CCNB1 pathway may be involved in RP11-295G20.2-driven HCC progression.

There are several limitations in the current study. First, only data from TCGA database was analyzed to assess the prognostic value of RP11-295G20.2 expression in patients with HCC, but we did not employ the clinical data from our center to further validate the results of bioinformatics analyses based on TCGA data. Second, we explored the functions of RP11-295G20.2 in Hep3B and MHCC-97H cells and the potential molecular mechanisms, but the effects of RP11-295G20.2 on the other HCC cell lines have not been evaluated. Third, the function of RP11-295G20.2 was explored only through downregulating RP11-295G20.2 expression in a loss-of-function model. However, Gain-of-function studies via overexpression of RP11-295G20.2 in HCC cells are required to confirm our findings. In future, more experiments should be conducted to resolve these limitations, which will further largely support the notion that RP11-295G20.2 acts as an oncogene in HCC progression.

In conclusion, this study revealed that RP11-295G20.2 can promote HCC progression at least partly via the miR-6884-3p/CCNB1 pathway, suggesting that RP11-295G20.2 may be a potential target for HCC therapy.

## MATERIALS AND METHODS

### Clinical tissues collection

A total of 30 pairs of HCC samples and matched adjacent normal liver tissue samples were collected from the patients who underwent liver resection for HCC at The Third affiliated Hospital of Sun-Yat sun University. None of the patients had received any other anti-tumor therapy before surgery. The study protocol was approved by the Ethics Committee of The Third Affiliated Hospital, Sun-Yat sun University. Moreover, written informed consent was provided by all the participants.

### Cell culture and transfection

Four kinds of HCC cell lines (HepG2, Hep3B, Huh7 and MHCC-97H) and one normal hepatocyte cell line (L0-2) were purchased from American Type Culture Collection (Manassas, VA, USA). Cell lines were cultured in DMEM supplemented with FBS and were maintained at 37 °C in a humidified incubator supplied with 5% CO2. HCC cells transfected with small interfering RNA (siRNA) targeting RP11-295G20.2 (si-RP11-295G20.2) and negative control siRNA (si-NC) synthesized by Guangzhou Ribobio Co., Ltd. (Guangzhou, China) were used for the in vitro experiments. The shRNA targeting RP11-295G20.2 (sh-RP11-295G20.2) and its scrambled shRNA were transfected into HCC cells using lentivirus method to construct cells with RP11-295G20.2 stably silenced and theses cells were subjected for the in vivo experiments. The miR-6884-3p mimics, NC miRNA mimics (miR-NC), miR-6884-3p inhibitor, and NC inhibitor were obtained from Shanghai GenePharma Co., Ltd. (Shanghai, China). To restore CCNB1 expression, the full-length sequence of CCNB1 without the 3-UTR was established by Guangzhou GeneCopoeia Co., Ltd. (Guangzhou, China) and transfected into the pcDNA3.1 vector. The resulting construct was named as pcDNA3.1-CCNB1 (pc-CCNB1).

### RNA isolation and real-time quantitative PCR (qRT-PCR)

Total RNA was isolated from the tissues or cultured cells by means of the TRIzol reagent (Invitrogen; Thermo Fisher Scientific, Inc., Waltham, MA, USA). To quantify miR-6884-3p expression, we used the miScript Reverse Transcription kit (Qiagen GmbH, Hilden, Germany) for the preparation of complementary DNA (cDNA) from total RNA. Then, we amplified the cDNA using the miScript SYBR Green PCR kit (Qiagen GmbH, Hilden, Germany) on a Roche Light Cycler 480 Real-Time PCR System (Roche Diagnostics, Basel, Switzerland). Relative miR-6884-3p was normalized to that of small nuclear RNA U6. To determine the expression of RP11-295G20.2 and CCNB1 mRNA, we conducted reverse transcription using the PrimeScript RT Reagent Kit (Takara Biotechnology Co., Ltd., Dalian, China). Next, quantitative PCR was conducted using the SYBR Premix Ex Taq™ Kit (Takara Biotechnology Co., Ltd., Dalian, China). GAPDH was regarded as the endogenous control to normalize the expression levels of RP11-295G20.2 and CCNB1 mRNA. All data were analyzed by the 2^–ΔΔC^t method. The primers for RT-qPCR are as following: CCNB1 (Forward: AATAAGGCGAAGATCAACATGGC; Reverse: TTTGTTACCAATGTCCCCAAGAG); GAPDH (Forward: GGAGCGAGATCCCTCCAAAAT; Reverse: GGCTGTTGTCATACTTCTCATGG); RP11-295G20.2 (Forward: CGAACAAAGGAACAGTAAATGG; Reverse: ACATGCCTTGTAGTTACTGC).

### Cell counting kit-8 (CCK-8) assay

The proliferative capacity of HCC cells was evaluated using CCK-8 assay. After transfected for 24 hours, the cells were collected. Then, they were suspended and seeded in 96-well plates at a density of 3,000 cells per well. Transfected cells were then maintained at 37 °C in a humidified incubator supplied with 5% CO_2_ for 0, 24, 48, or 72 hours. The CCK-8 assay was conducted at every time point by adding 10 μL of the CCK-8 reagent (Dojindo Molecular Technologies, Inc., Kumamoto, Japan) into each well. After incubated for 2 hours, the optical density (OD) of each well at a wavelength of 450 nm was measured on a microplate reader (Bio-Rad Laboratories, Hercules, CA, USA).

### Colony formation assay

HCC cells were harvested 24 hours after transfection and seeded in 6-well plates at a density of 1000 cells/well. The cells were kept at 37°C in a humidified incubator supplied with 5% CO_2_ for 2 weeks. Next, we fixed the cells using 95% methanol and then stained them with methyl violet (Beyotime Institute of Biotechnology, Inc., Shanghai, China). The tumor colonies (>50 cells) were counted under an IX71 inverted microscope (Olympus Corporation, Tokyo, Japan).

### Cell apoptosis assay

The percent of apoptotic cells was tested using an Annexin V–Fluorescein Isothiocyanate (FITC) Apoptosis Detection Kit (Biolegend, San Diego, CA, USA). The HCC cells were incubated for 48 hours and harvested. Next, we washed the collected cells for three times using cold phosphate-buffered saline (PBS) and then resuspended them in 100 μL of binding buffer. After that, we put 5 μL of Annexin V–FITC and 5 μL of a propidium iodide solution to the HCC cells and kept them at room temperature in the dark environment for 15 minutes. At last, we determined the percent of apoptotic cells using flow cytometry (FACScan; BD Biosciences, Franklin Lakes, NJ, USA).

### Transwell migration and invasion assays

Transwell chambers (8.0 μm pore size; BD Biosciences, Franklin Lakes, NJ, USA) covered with Matrigel (BD Biosciences, Franklin Lakes, NJ, USA) were applied to conduct the invasion assay. Transwell chambers without Matrigel were used for the migration assay. A total of 5 × 10^4^ transfected HCC cells were suspended in 200 μL of FBS-free DMEM and then added into the upper compartment of the Transwell chambers. Meanwhile, a total of 500 μL of DMEM supplemented with 20% of FBS was used as a chemoattractant, which was added into the lower compartments. After 24 hours, HCC cells that still stayed on the upper surface of the membranes were carefully erased using a cotton swab. The HCC cells that migrated and invaded into the other surface of the membranes were fixed using 95% ethanol and stained using 0.5% crystal violet (Beyotime Institute of Biotechnology, Inc., Shanghai, China). At the last step, we counted the number of migrated and invaded cells in five randomly selected visual fields from each chamber using an inverted microscope.

### RNA immunoprecipitation (RIP) assay

The binding of miR-6884-3p to RP11-295G20.2 was identified using the Magna RIP RNA-Binding Protein Immunoprecipitation Kit (Millipore Inc., Billerica, MA, USA). After lysing HCC cells with RIP buffer, we collected the cell extract and incubated it using magnetic beads conjugated with a human anti-AGO2 antibody or control IgG (Millipore Inc.). Next, we removed the protein of the collected samples using proteinase K and then isolated the total RNA for RT-qPCR analysis.

### Bioinformatic analyses

Survival information of 364 patients with HCC from TCGA data was analyzed to evaluate the association between RP11-295G20.2 expression and overall survival. LncBase v.2 and RNAhybrid was employed to search for the miRNAs that may be sponged by RP11-295G20.2. The putative targets of miR-6884-3p were predicted using TargetScan (http://www.targetscan.org/).

### Luciferase reporter assay

The 3′-UTRs of RP11-295G20.2 or CCNB1, which contained the wild-type sequence (with a specific binding site for miR-6884-3p) or mutated sequence (without a binding site for miR-618), were cloned into a luciferase reporter vector (GeneChem, Shanghai China). Cells were co-transfected with one of the luciferase reporter plasmids, miR-6884-3p mimics, and oligonucleotides (NC) for 48 hours. Finally, the cells were collected and subjected to a dual-luciferase reporter assay (Promega, Madison, WI, USA) to assess the Firefly and Renilla luciferase activities.

### Western blot analysis

Expression of CCNB1 protein was detected by Western blotting assay. We firstly extracted the total protein using a ProteoPrep ® total extraction sample kit (Sigma-Aldrich). Next, a total of 80 μg of protein extract was separated by SDS-PAGE and then electrophoretically transferred onto nitrocellulose membranes. After that, we blocked the nitrocellulose membranes with using 5% skim milk for one hour, and then incubated them with rabbit anti-CCNB1 (1:1000 dilution, CST) at 4°C overnight. Washed three times by TBST, the membranes were incubated using HRP-labeled goat anti-rabbit IgG (1:500 dilution, Santa Cruz) for another one hour. Besides, GAPDH was used as an endogenous control. Protein bands were visualized with an ECL detection kit (Amersham Pharmacia Biotech, NJ, USA) and quantified using ImageJ software 1.8(National Institutes of Health, Bethesda, USA).

### Statistical analysis

We collected all data from three independent experiments and they were presented as the mean ± SD. We performed all the statistical calculations using GraphPad. Two-tailed Student’s t-test or Chi-square test was carried out to determine the differences between two groups. The correlation of miR-6884-3p with RP11-295G20.2 or CCNB1 expression levels in HCC tissues was evaluated by Spearman’s correlation analysis. The survival curve was constructed by the Kaplan–Meier method and survival was compared using the log-rank test. P < 0.05 was considered as statistical significance.

### Ethics approval

In this study, all procedures associated with patients and animals have been approved by the ethical standards of The Third Affiliated Hospital of Sun-Yat sun University. Informed consent to participate in this study has been obtained from participants.

## Supplementary Material

Supplementary Figure 1
