# Supplementary material for: RP11-295G20.2 facilitates hepatocellular carcinoma progression via the miR-6884-3p/CCNB1 pathway
Source: Aging (Albany NY). 2020 Jul 20;12(14):14918–32. doi: 10.18632/aging.103552 (PMC7425504; doi:10.18632/aging.103552)
Supplement: Supplementary Figure 1 [file aging-12-103552-s001..pdf]

SUPPLEMENTARY FIGURE

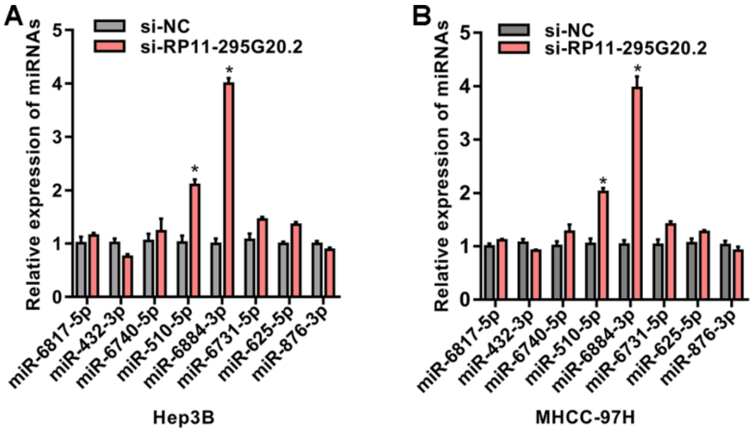

Supplementary Figure 1. The expression levels of miR-6817-5p, miR-432-3p, miR-6740-5p, miR-510-5p, miR-6884-3p, miR-6731-5p, miR-625-5p, and miR-876-3p following transfecting si-NC or si-RP11-295G20.2 in Hep3B cell lines (**A**) or MHCC-97H cell lines (**B**),*P < 0.05.
